# Cadmium Protection Strategies—A Hidden Trade-Off?

**DOI:** 10.3390/ijms17010139

**Published:** 2016-01-21

**Authors:** Adolf Michael Sandbichler, Martina Höckner

**Affiliations:** University of Innsbruck, Institute of Zoology, Technikerstraße 25, 6020 Innsbruck, Austria; adolf.sandbichler@uibk.ac.at

**Keywords:** cadmium, protection, detoxification, antioxidants, chelation, metallothionein, hormesis, oxidative stress, mitochondrial damage, nuclear response factor 2 signaling

## Abstract

Cadmium (Cd) is a non-essential transition metal which is introduced into the biosphere by various anthropogenic activities. Environmental pollution with Cd poses a major health risk and Cd toxicity has been extensively researched over the past decades. This review aims at changing the perspective by discussing protection mechanisms available to counteract a Cd insult. Antioxidants, induction of antioxidant enzymes, and complexation of Cd to glutathione (GSH) and metallothionein (MT) are the most potent protective measures to cope with Cd-induced oxidative stress. Furthermore, protection mechanisms include prevention of endoplasmic reticulum (ER) stress, mitophagy and metabolic stress, as well as expression of chaperones. Pre-exposure to Cd itself, or co-exposure to other metals or trace elements can improve viability under Cd exposure and cells have means to reduce Cd uptake and improve Cd removal. Finally, environmental factors have negative or positive effects on Cd toxicity. Most protection mechanisms aim at preventing cellular damage. However, this might not be possible without trade-offs like an increased risk of carcinogenesis.

## 1. Introduction

Over the last decades, several studies have looked into the toxic effects of cadmium (Cd) at cellular and organismic levels to assess the risk of increasing environmental pollution by heavy metals. Cd is a known carcinogenic and immunotoxic heavy metal. An estimated 30,000 tons of Cd are released into the environment each year. Cd is highly persistent in the environment and also enters the food chain [1]. Cd toxicity is mainly based on so-called ionic mimicry which is defined by the replacement of elements like calcium (Ca^2+^) and trace elements like zinc by Cd^2+^ [2]. This can lead to protein mis- or unfolding and malfunction and eventually cause endoplasmic reticulum (ER) stress and cell death [3].

The induction of oxidative stress appears to be another indicator of the damaging mechanism of Cd as shown by a considerable body of evidence. This is caused indirectly, as Cd is not a redox active metal, through the depletion of the cells’ major antioxidants and direct interference with active centers of the electron transport chain [4,5,6]. We discuss the protective measures employed at the cellular and organismic level when confronted with Cd. With this change in perspective from “what is damaged” to “how detrimental effects can be overcome or even bypassed”, this review discusses protection strategies against Cd insult. We focus on the effect of Cd at the cellular level including results from *in vivo* studies where novel defense mechanisms are presented but detailed cellular explanations have yet to be found.

Available defense strategies against Cd are grouped according to their underlying mechanisms. These include antioxidant defense, mitochondrial protection, metal chelation, prevention of macromolecular damage, cytoskeletal rearrangements, hormetic response, co-exposure to other metals or trace elements, reduced uptake of Cd, removal of Cd, and toxicity of Cd altered by environmental factors.

In the current review, we summarize the variety of protective responses against Cd insult which are based on highly diverse mechanisms. However, when implemented, most of these defense strategies contain trade-offs like anti-apoptotic effects and risk of carcinogenesis.

## 2. Results and Discussion

### 2.1. Protection via Antioxidants

Cd is not able to produce radicals in Fenton type chemistry. Nonetheless, it induces oxidative stress through a multifaceted mechanism including the reduction of antioxidative defense and the production of reactive oxygen species (ROS) by mitochondrial damage (see Section 2.2).

Upon entry into the cell, Cd forms complexes with thiol residues from the tripeptide-reduced glutathione (GSH), the main intracellular antioxidative substance. GSH complexation with Cd^2+^ (termed GS-Cd) is considered a first line of defense since it prevents the heavy metal from causing further damage and in some cases enables active removal through specialized transporters (see Section 2.9) [7,8,9]. Due to the reduction of free GSH levels by Cd^2+^ binding, the cells redox balance is shifted to a more oxidized state and antioxidative defense is impaired. Interestingly, only recently a study on rat proximal tubule cells has shown the induction of GSH synthase subunit genes. As a protective response to Cd intoxication, GSH synthase recycles oxidized glutathione [10]. The same study also tested for chronic effects *in vivo* and found elevated gene expression for catalase (CAT), mitochondrial superoxide dismutase 2 (SOD), glutathione peroxidase 4, and peroxiredoxin 2 after daily subcutaneous Cd injections.

A second important redox system besides GSH/oxidized glutathione (GSSG) is the thioredoxin (Trx) system. The central enzyme Trx reductase (TrxR), a selenoprotein which recuperates reduced Trx using nicotinamide adenine dinucleotide phosphate (NADPH), can be induced by Cd to evoke a protective response. In bovine arterial endothelial cells, such Cd-induced expression of TrxR isoform 1 was mediated by nuclear response factor 2 (Nrf2) which binds to an antioxidative response element (ARE) in the promotor region of TrxR1 [11].

Other examples for the induction of antioxidative enzymes via ARE binding of Nrf2 include hemeoxygenase-1 and glutamate-cysteine ligase [12] or SOD [13]. 

Different natural compounds and phytochemicals have protective potential in Cd intoxication (Table 1). Many of the compounds tested are referred to as “natural antioxidants” but actually function as activators of Nrf2 leading to the upregulation of the antioxidant machinery [14]. Given these observations it is not surprising that Nrf2 signaling is believed to be an important regulator of cellular resistance to oxidants [15]. Indeed, upregulation of Nrf2 has also been shown to have negative effects: A growing body of evidence finds that cancer cells employ this mechanism to raise their resistance to oxidative stress, reprogram metabolism, and sustain cell proliferation [14]. Interestingly, Cd itself has only weak genotoxic effects but secondary carcinogenic effects and tissue damage can occur by way of oxidative stress [6,16,17,18]. Such carcinogenic damage can be reduced by a number of natural antioxidants (Table 1). However, if this includes Nrf2 activation, short-term amelioration of Cd-induced ROS may lead to carcinogenic effects in the long term. Ultimately, due to the direct inhibition of DNA repair enzymes such as the human 8-oxoguanine DNA *N*-glycosylase (hOGG1) by Cd, the carcinogenic potential of Cd is even potentiated by DNA changes [18,19].

**Table 1:** Protective natural compounds and phytochemicals against Cd intoxication.

Substance	Source	[Cd]/Duration/Experimental Animal	References
Curcumin ^a,b^	Turmeric (*Curcuma longa* L.)	24 h Cd exposure, *in vivo*, rodents *In vitro*, human airway epithelial cells	[20,21,22,23]
Ginger	Ginger (*Zingiber officinale*)	200 mg/kg b.w., 12 weeks, *in vivo*, rabbits	[24]
Resveratrol ^b^	Polyphenol from skin of grapes (*Vitis vinifera*)	7 mg/kg b.w., 24 h exposure, *in vivo*, mice	[21]
Physalis extract	*Physalis peruviana* L.	6.5 mg/kg b.w., 5 days, *in vivo*, rats	[25]
Grapefruit juice ^a^	Grapefruit	1.5 mg/kg b.w., from day 7 of gestation until day 17 of pregnancy, *in vivo*, mice	[26]
Garlic extract or Allicin ^b^	Garlic	5 or 10 ppm, 45 days, *in vivo*, Freshwater catfish (*Clarias batrachus*)	[27]
Royal jelly ^a^	from Honey bees	2 mg/kg b.w., 6–7 weeks, *in vivo*, mice	[28]
Spirulina ^a^	Micro-algae spirulina (*Arthrospira maxima*)	1.5 mg/kg b.w., 1 time Cd challenge, *in vivo*, pregnant mice; 3.5 mg/kg b.w., 1 time Cd intraperitoneal dose, *in vivo*, rats	[29,30]
Farnesol ^a^	Isoprenoid from aromatic plants	5 mg/kg b.w., 1 time Cd, *in vivo*, mice	[31]
Theaflavin	Polyphenol from black tea (*Camellia sinensis*)	0.4 mg/kg b.w., once a day, for 5 weeks, *in vivo*, rats	[32]
Taxifolin	Bioflavonoid from conifers	100 μM Cd, *in vivo*, Zebrafish (*Danio rerio*)	[33]
Quercetin	Bioflavonoid from apples and onions	4 mg/kg b.w. for 2 weeks, *in vivo*, mice; 1.2 mg Cd/kg/day, 5 times/week during nine weeks, *in vivo*, rats 5 µM, *in vitro*, in cultured granulosa cells from chicken ovarian follicles	[34,35,36]
Naringenin	Bioflavonoid from grapefruit	5 mg/kg, orally for 4 weeks, *in vivo*, rats	[37]
Rosemary extract ^b^	*Rosmarinus officinalis* L.	30 mg/kg b.w., 5 consecutive days/week for 8 weeks, *in vivo*, rats	[38]
Catechin ^a,b^	Polyphenol from Green tea (*Camellia sinensis*)	50 ppm *ad libitum*, 20 weeks, *in vivo*, rats	[39]
Sulforaphane ^a,b^	Isothiocyanate from cruciferous vegetables	*In vitro* in human hepatocytes and *in vivo* in mice; 0.2 mg/kg, 15 days, *in vivo*, rats	[40,41]

^a^ shown to prevent Cd-induced genotoxic effects; ^b^ suspected to induce nuclear response factor 2 (Nrf2) signaling [14,42]; b.w. body weight.

Numerous studies have already shown the protective role of hormones like melatonin [43,44,45], antioxidative vitamins [27,40,46,47,48,49], and antioxidants such as *N*-acetylcysteine (NAC) [50,51,52,53,54].

**Figure 1:**
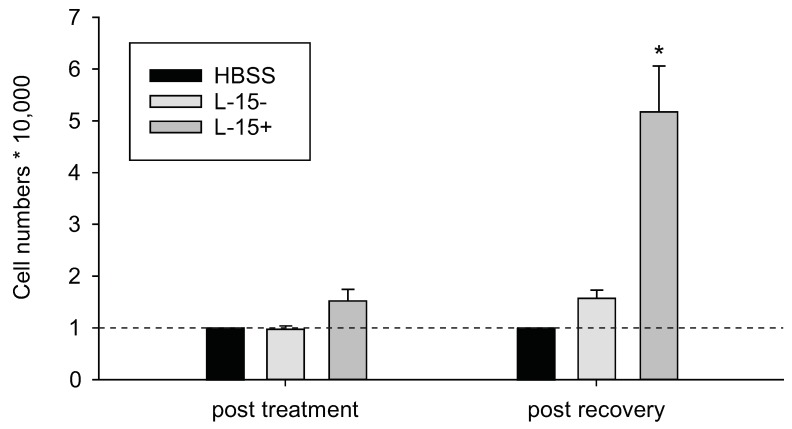
Cell density assay (Hoechst 33342) with Z3 zebrafish cells in control experiments using different culture media. Cell numbers were measured once after the 18 h treatment and once after the 6 h recovery period. L-15−: L-15 complete media without FBS L-15+: L-15 complete media. Cell numbers were normalized to 10,000 cells of the Hank’s buffered salt solution (HBSS) control. Statistical analysis was performed prior to data normalization using a *t*-test. Groups were compared to HBSS treatment (* *p* ≤ 0.05). Values are mean ± standard error from 3 biological replicates.

In the following, we show different effects of two antioxidants, ascorbic acid (vitamin C, VC) and NAC, on Cd-impaired cell survival in a zebrafish embryonic fibroblast cell line (Z3) in order to assess the potential and putative differences of VC and NAC in the recovery from Cd-induced oxidative stress. Z3 cells were serum-deprived by incubation in Hank’s buffered salt solution (HBSS), which is known to induce ROS and eventually lead to apoptosis [55]. In fact, cell density in HBSS-treated cells but also cells treated with cell culture media lacking fetal bovine serum (FBS) was decreased compared to cells incubated with complete media (Figure 1).

There is no general agreement on the effect of NAC on Cd toxicity since several studies suggest a protective effect on cell viability, e.g., in rat hepatocytes, when cells were co-exposed to Cd and NAC [53]. However, another study observes a cumulative toxic effect of NAC and Cd. In human HaCaT cells, only pre-treatment with NAC restored Cd-induced cell death which led the authors to the conclusion that Cd and NAC might form complexes with one another or with the culture media [56]. In Leydig cells, NAC pre-treatment also revealed decreased cell death via the reduction of oxidative damage [57], and in HepG2 cells, Cd-induced apoptosis could be reduced by NAC-dependent upregulation of catalase [58]. Another study reveals that NAC changes the expression of cytokines and chemokines and suggests that the immunomodulatory effect protects against Cd toxicity [59]. Studies on the protection mechanisms of NAC reveal that NAC increases phosphorylated p38 MAPK by decreasing the ROS level in a human osteosarcoma cell line [60]. Similarly, it has been found that in zebrafish embryo NAC protects against msh6 inhibition which is part of the DNA mismatch repair, most likely also by decreasing ROS [61].

**Figure 2:**
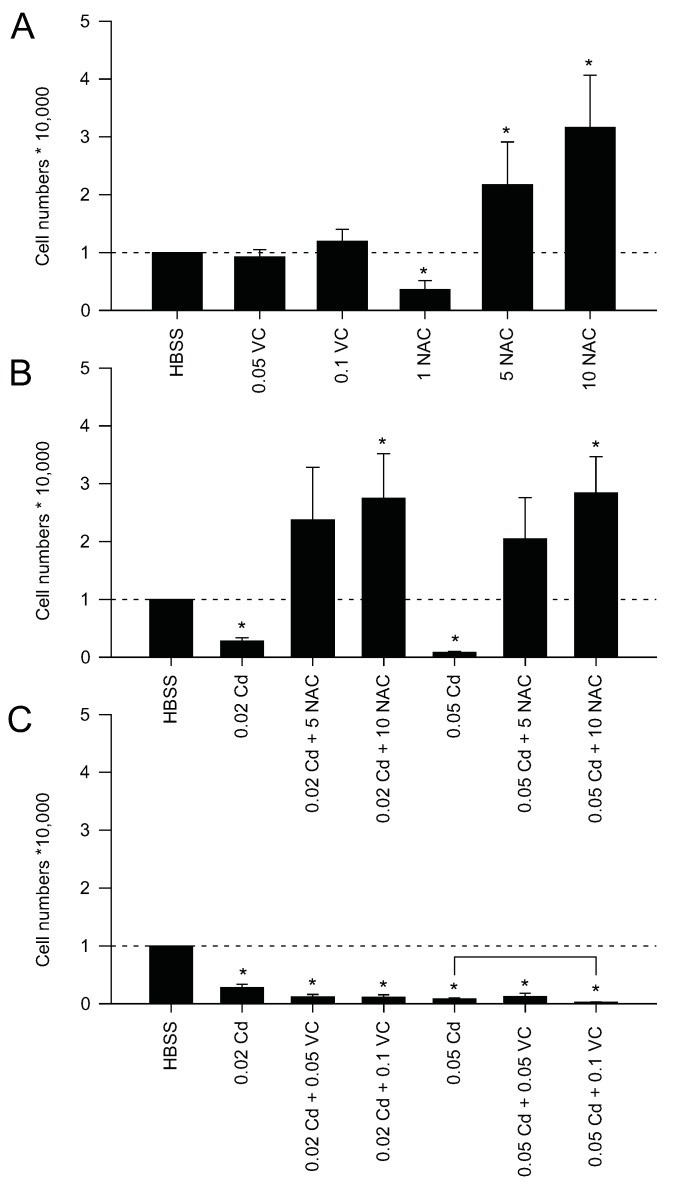
Cell density assay (Hoechst 33342) with Z3 zebrafish cells. (**A**) Effect of vitamin C (VC) and *N*-acetylcysteine (NAC) on HBSS incubated cells; (**B**) Recovery from HBSS and Cd treatment using NAC; (**C**) Recovery from HBSS and Cd treatment using VC. Cell numbers were normalized to 10,000 cells of the HBSS control. Statistical analysis was performed prior to data normalization using a *t*-test. Exposures were compared to HBSS treatment (* *p* ≤ 0.05). Square bracket indicates statistical significance from comparison of normalized data. Values are mean ± standard error from 3 biological replicates.

Original data included in the present review article reveal that NAC is able to restore cell numbers of Z3 zebrafish cells upon HBSS starvation and Cd exposure (Figure 2). The experiments were conducted in HBSS to overcome putative problems caused by the formation of complexes between Cd^2+^ and components of the cell culture media, as stated above. We, therefore, conclude that NAC protects against Cd-induced oxidative stress via its antioxidant capacity affecting cellular mechanisms which might differ between cell types and tissues.

Interestingly, VC was, in contrast to NAC, not able to restore cell numbers upon HBSS starvation in Z3 zebrafish cells (Figure 2). Co-exposure to HBSS, CdCl_2_, and VC even caused a cumulative toxic effect further decreasing Z3 cell numbers (Figure 2C). Preparation of the treatment solutions in HBSS excludes complex formation with cell culture media components, so we suggest that VC and CdCl_2_ form compounds with higher toxicity than Cd alone or that VC increases or facilitates Cd uptake by Z3 cells. As shown before, Cd is responsible for δ-aminolevulinate dehydratase enzyme inhibition in rat lung and VC even increased the inhibiting effect [62]. However, VC has also been shown to attenuate germ cell apoptosis by protecting against ER stress and unfolded protein response (UPR) in mouse testes [63]. According to another study, VC inhibits lipid peroxidation in rat testes [46]. VC has also been shown to protect against Cd-induced renal injuries [64] and to reduce Cd accumulation in liver and kidney of catfish [27].

Taken together, the present results from a zebrafish cell line and many previous studies show that NAC and VC have different effects on Cd toxicity. The impact of NAC and VC might, therefore, be cell type and tissue-specific; underlying mechanisms, however, remain to be resolved.

In conclusion, it can be stated that a major protection mechanism against Cd-induced ROS can be found in the induction and upregulation of the intrinsic antioxidative machinery. Indeed, several studies employ the idea of assaying the induction of oxidative response as a biomarker for Cd contamination, for example in Nile tilapia [65] and bivalves [66,67,68]. Furthermore, it can be postulated that many different means able to reduce oxidative stress will also ameliorate Cd-induced toxicity although this may be cell and tissue-specific and might also have negative effects.

### 2.2. Mitochondrial Protection Counteracts Cd Insult

A well-studied detrimental effect of Cd exposure is mitochondrial damage due to increased ROS levels but also deviations in Ca^2+^ homeostasis [69,70,71]. Since mitochondria are important Ca^2+^ stores inside the cell, Cd^2+^ leads to a competitive inhibition of calcium translocation and homeostasis. Consequently, ROS and Ca^2+^ disturbance lead to numerous changes in the mitochondrial status including the reduction of oxidative phosphorylation, depolarization of mitochondrial membrane potential (ΔΨ_m_), increase of superoxide and decrease of ATP production [72], and, ultimately, to mitophagy [73,74]. The mitochondria-specific increase in ROS was shown to be caused by direct interaction of Cd with the electron transport chain (ETC) [75].

The exact sequence of mitochondrial degradation caused by Cd has been demonstrated along different lines. Some studies show Cd-induced opening of the mitochondrial permeability transition pore (mPTP) [75,76,77]. According to another study on rat proximal tubule cells, Cd^2+^ enters mitochondria via the mitochondrial calcium uniporter (MCU) and induces mPTP-independent swelling of mitochondria [78]. However, both mitochondrial dysfunctions caused by Cd, mPTP and Cd entry via the MCU, lead to the release of cytochrome C from the intermembrane space into the cytosol, an important step in the initiation of apoptosis.

In summary, mitochondria represent a central target for Cd-induced toxicity and different means of mitochondrial protection against Cd toxicity apply. When considering, for example, normal mitochondrial turnover which is tightly controlled by fission and fusion rates of mitochondrial fragments, we can postulate that prevention of stress-induced mitochondrial fragmentation should protect mitochondria against Cd. Indeed, a recent study has shown that silencing a central fission-promoting protein (Drp1) reduces Cd-induced mitophagy [79].

Clearly, Cd toxicity in mitochondria is often based on oxidative stress and most of the afore mentioned detrimental effects such as depolarization of ΔΨ_m_, mPTP, swelling or fission would also occur under ROS stress, for example as a result of hypoxia/reoxigenation. Therefore, antioxidative intervention is able to protect mitochondria against Cd insult. Both, pyruvate, known to protect against oxidative stress [72], and melatonin, known for its effects on free radical scavenging [80], have been shown to directly protect mitochondria. Examples of natural antioxidative substances or substances activating antioxidative defense under Cd exposure are listed in Table 1.

While different pathways of Cd-induced mitochondrial damage have been studied in recent years, it remains unknown how cells protect themselves and their mitochondria against heavy metal insult. Remarkably, several protective options exist. A recent study has found a positive induction of mitochondrial biogenesis and mitochondrial DNA content after acute Cd toxicity in rat proximal tubule cells as well as after chronic exposure *in vivo* [10]. The authors also found a distinct upregulation of anti-apoptotic genes with chronic exposure. This result may indicate an attempt to counteract Cd-induced apoptosis triggered by mitochondria and/or ER. Another study identified an upregulation of the mitochondrial NADP+-dependent enzyme isocitrate dehydrogenase to ameliorate oxidative stress by providing NADPH which serves as a reduction equivalent for the regeneration of GSH [81].

Another protective effect involving mitochondria can be observed in the Cd-induced downregulation of metabolism itself. Dogwhelks, aquatic gastropods, cope with a 20-day Cd exposure by metabolic depression. This physiological adaptation is a common response to intermittent hypoxia but it could also be shown to occur under Cd stress. The authors claim this response to be a strategy to minimize Cd^2+^ uptake and meet the extra energy demand for detoxification [82]. It is tempting to ask whether this is merely an effect of oxidative stress and mitochondrial damage or truly a protection mechanism. Indications can be drawn from a recent publication studying energy utilization of mitochondria in the freshwater crab *Sinopotamon henanense*. These experiments show that mitochondria respond to acute Cd exposure with an upregulation of energy production (higher levels of ΔΨ_m_, NADH/NAD+ and ATP/ADP ratio) to cope with the energy demand of cellular defense mechanisms such as metallothionein (MT) production. However, with increasing exposure time a decline of energy production accompanied by excessive mitochondrial impairment was observed [83]. Consequently, it can be stated that mitochondrial energetic homeostasis is a fundamental requirement for successful Cd defense [83,84] but long-term countermeasures may depend on a balanced mitochondrial turnover with the risk of emphasizing anti-apoptotic signaling.

### 2.3. Protection by Metal Chelation

One of the major detoxification mechanisms protecting the cell from Cd-induced damage is the direct binding of Cd^2+^ to metal chelators. Among the most important and well-studied Cd^2+^-binding proteins are MTs [85]. MTs occur throughout the animal kingdom and are involved in diverse cellular tasks including antioxidative functions [86,87]. However, their main responsibility is the homeostasis and detoxification of metals. Several MT isoforms have been described, the numbers differ within species with 12, *i.e.*, most, being present in mammals. The first *in vivo* Cd^2+^ binding studies using mouse MT1 were performed in the late 90s suggesting that domain duplication events in MTs might have evolved to not only function in trace metal homeostasis but also to cope with toxic metals like Cd [88]. In terrestrial gastropods, the evolution of a MT isoform showing an extraordinary Cd^2+^-binding specificity was observed [89]. A recent study on mammalian MT1A revealed that the domain-specific order of the binding reaction and not the binding affinities account for the binding of zinc or Cd^2+^ [90], whereas it had already been shown that MT1 is more significantly sequestering Cd^2+^ than MT2 [91]. Several examples show that MT isoforms evolved to take over isoform-specific functions like Cd detoxification in mollusks [92,93], sea urchins [94], *Drosophila* [95], *C. elegans* [96], and *Tetrahymena* species [97,98]. However, due to its angiogenic, anti-apoptotic and proliferative functions, MT upregulation has been connected with poor prognosis and increased chemotherapeutic resistance [99,100] in some types of cancer.

Combined with the fact that MT gene expression can be directly induced by Cd, it can be stated that this mechanism presents one of the most efficient and prominent protection strategies against Cd. In vertebrates and insects, the metal transcription factor 1 (MTF-1) is responsible for MT induction. In the presence of Cd, MT-bound zinc is replaced by Cd^2+^ which is then able to activate MTF-1 [101]. Then again, except for insects, the MT activation mechanisms in invertebrates might be regulated [102] differently, in earthworms probably via the cAMP response element (CRE)-binding protein [103].

MTs are mainly expressed in the liver where Cd-MT complexes are formed. A thorough overview of structural characterization and binding affinities of Cd^2+^ in MTs can be found elsewhere [104]. Initially, Cd-MT complexes are stored in lysosomes, but are released into the bloodstream once liver cells die off. In colonic epithelial cells the uptake of Cd-MT complexes and their translocation to lysosomes lead to a decrease of systemic Cd toxicity [105]. However, Cd-MT complexes might still bear the risk of cellular damage. This has been shown in a study using a rat ADP ribosylation factor 1 (Arf1) mutant (Arf1 is involved in late endosome/lysosome trafficking) which decreased Cd toxicity in renal cells probably by attenuating the release of Cd^2+^ from degraded MT1 complexes into the cytosol [106]. The kidney is also known to be severely affected by Cd exposure. According to a recent study, Cd^2+^ causes hyperpermeability and hence disrupts the endothelial cell barrier in the glomerulus [107]. Due to its low molecular weight, the Cd-MT complex is filtered out at the glomerulus and is incorporated into proximal tubular cells. Subsequently, this can lead to kidney injuries. However, if the receptor responsible for Cd-MT incorporation is inhibited, Cd-MT-induced toxicity is reduced in the kidney [108] (see Section 2.8). Taken together, MT is pivotal in the protection against Cd-induced toxicity but also plays a central role in the systemic cycling of Cd and may hold carcinogenic potential due to its diverse functions.

Glutathione, which has already been described as an antioxidant, also acts as a metal-chelating agent able to bind Cd [109]. In addition, GSH is involved in cellular removal of Cd and is discussed later. Phytochelatins (PCs), which are formed from condensation of glutathione molecules, have recently been discovered in invertebrate species [110] and are also believed to function as a Cd detoxification system [111]. In contrast to Cd-MT, Cd-PC complexes taken up with the food have been shown to not co-localize with lysosomes [105] which might hint at different storage and excretion routes of PCs and MTs.

Cd chelation via MT, GSH, and PC represents a highly efficient detoxification system. However, a putative degradation of the metal-protein complex may lead to a repeated release of toxic Cd ions.

### 2.4. Protection against Macromolecular Damage

The endoplasmic reticulum (ER) is the major Ca^2+^ store inside the cell. It is, therefore, not surprising that Cd intoxication involves ER stress by altering Ca^2+^ homeostasis [112]. Moreover, the ER is the site for protein folding and refolding, which also play a major role in Cd toxicity. Since Cd^2+^ has a similar hydration radius like Ca^2+^, it enters the cell through Ca^2+^ channels, interacts with Ca^2+^ pumps in the ER membrane and damages the ER upon entry [113]. Furthermore, Cd^2+^ is structurally very similar to essential trace elements like zinc. This ionic mimicry is responsible for protein misfolding or malfunction. Therefore, the ER is not only challenged directly by altered Ca^2+^ levels but also by an increase of damaged proteins. The cellular response to ER stress can involve adaptive mechanisms which protect the cell against stress or can lead to Cd-induced apoptosis. Several proteins have been found to be involved in mediating between cell survival and cell death. However, the point of no return has not yet been identified [114]. An indicator of ER stress is the upregulation of the unfolded protein response (UPR) which can activate pro-survival signals or induce apoptotic cell death. Several types of tumors depend on this mechanism, because several branches of the UPR positively affect cell transformation and tumor aggressiveness [115]. A strategy to reduce macromolecular damage causing ER stress and subsequent UPR is the expression of chaperones like Grp78. Grp78 is located in the ER and is known to be induced upon Cd exposure to prevent protein unfolding or misfolding as shown in LLC-PK1 renal epithelial cells [116].

Regarding ER stress and Cd intoxication, it could also be shown that once again Nrf2 [117], ubiquitin ligase FBXO6 [118] as well as ascorbic acid [63], a well-known antioxidant, attenuate Cd-induced ER stress. In concordance with the latter, the prevention of ER stress in Cd-resistant cells is responsible for cell survival via the activation of p38 and the induction of autophagy [119].

The heat shock response represents a general protective mechanism against environmental stress and specifically against Cd exposure via an increased expression of heat shock proteins (HSPs). HSPs represent cytosolic chaperones involved in protein folding and in the antioxidant response. The protective role of HSPs in Cd toxicity might be exerted via ROS scavenging [120]. So far, a time-dependent induction of HSPs upon Cd exposure has been revealed [121]. However, Cd-induced reduction of FcHsp70 was observed in the Chinese shrimp *Fenneropenaeus chinensis* [122], the Pacific oyster *Crassostrea gigas* [123], and in a human myeloid cell line [124]. In addition, the mRNA and protein level of HSPs can also differ as shown in the cyprinid fish *Tanichthys albonubes* [125].

In summary, the prevention and repair of molecular damage presents one of the major cellular tasks to maintain or re-establish homeostasis upon Cd exposure. In this context, ER stress prevention is an important protection mechanism in the short-term response to Cd administration but also bears the potential risk of carcinogenesis.

### 2.5. Cd Resistance and Cytoskeletal Rearrangements

As stated in the previous section, ionic mimicry, the competitive replacement of calcium ions by Cd^2+^, is a highly toxic mechanism for many cellular processes [2] such as the regulation of cytoskeletal elements through polymerization of the actin cytoskeleton [126]. Cd exposure has been shown to cause oxidation of peptidyl-cysteines in proteins regulating the actin skeleton [127] and epigenetic methylation of actin and myosin promotor regions in chinese hamster ovary cells [128]. Further studies have found F-actin depolymerization and apoptosis to be another effect of Cd^2+^—the chronological order of events is, however, still unknown [129]. Also, increased amounts of microtubules and microfilaments are able to protect a mouse cell line from Cd-induced damage by increasing the level of protein sulfhydryls. In the cytoskeletal and cytosolic fraction of Cd-resistant cells, the basal level of protein sulfhydryl groups was elevated. These cells show no cytoskeletal rearrangements upon Cd stress in contrast to parental cells [130]. Interestingly, in Cd-resistant rat lung epithelial cells, cytokeratins were upregulated, most likely to prevent Cd-induced apoptosis—a change in keratin expression is a highly probable protective response to long-term Cd exposure [131]. The involvement of Cd in malignant transformation of an immortalized cell line and the involvement of keratin was confirmed later [132]. Concluding, this protective mechanism also holds a potential trade-off in the form of carcinogenic transformation.

### 2.6. Protection against Cd by Cd—Hormetic Responses

Many terms have been used to describe beneficial dose-response relationships: hormesis, preconditioning, cross-resistance or adaptive protection. However, it has been suggested, that these phenomena all describe the same principle, namely the plasticity of biological processes and systems to adapt and respond to different kinds of stressors [133]. A simplified description of hormesis is the opposite dose-response relationship at low *versus* higher concentrations of a toxicant [134]. Accordingly, at low dosages, heavy metals can have a beneficial effect on the organism. A review of the mechanisms responsible for hormesis suggests that, regardless of the actual mechanisms involved, the intensity of the response is a measure of biological plasticity [135,136]. We, therefore, discuss the literature on the mechanisms underlying this biological plasticity to Cd exposure and its protective effects.

Cd has been shown to stimulate cell proliferation in zebrafish liver cells and to decrease the percentage of apoptotic cells by a change in expression of growth factors and DNA repair genes. Genomic instability might then, however, contribute to Cd-induced carcinogenesis [137]. Hormesis also induces other effects like the increase in cellular metabolic activity as shown in mouse fibroblast cells upon exposure to low levels of Cd which also coincided with an increased production of stress proteins like HSPs and MTs [138]. A study using HaCaT cells reveals that the proliferative response to low metal concentrations needs NADPH oxidase (NOX) stimulation which is activated by endogenous factors [139].

Hormetic effects of Cd were mainly studied at the organismic level. In adult rainbow trout (*Oncorrhynchus mykiss*), for example, chronic exposure to low dietary amounts of Cd decreases the toxic effect of waterborne Cd [140]. In mice, HSP70 and its activating heat shock factor 1 (HSF1) take over a major role in the protection and preconditioning to Cd administration [141]. In earthworms, hormetic effects upon Cd exposure affect antioxidant enzymes by increasing the activity of CAT and SOD [142]. Hormesis has also been described as a species-specific phenomenon. While exposure to small amounts of metals increased the rate of growth and reproduction in one species of snails, another species did not display any signs of hormesis [143].

It has also been shown that Cd induces cross-resistance to other metals like zinc [144] and manganese [145] or oxidative stress as shown in V97 Chinese hamster fibroblasts [9]. However, the cross-resistance effect does not seem to be bidirectional since stressors like oxidative stress can render cells more prone to a Cd challenge [146].

However, the beneficial effect of hormesis may not come without trade-offs. The exposure to dead spores causes longevity but also leads to reduced immune functions [147]. An additional stressor (depleted uranium) in the presence of radiation hormesis leads to an even higher toxicity (increased apoptosis) than the additional stressor alone would have caused [148]. It is important to note that the very ability of preconditioning can be deactivated by Cd exposure as demonstrated in a recent study in rats. The latter effect was attributed to the inhibition of hypoxia-inducible factor 1a (Hif1a) stabilization and the promotion of Hif1a degradation [149]. However, other authors show a clear induction of the Hif1a/vascular endothelial growth factor signaling axis by Cd [150].

In conclusion, beneficial effects derived from hormesis or hormesis-like phenomena should be critically reviewed especially when discussing the outcome at the organismic level.

### 2.7. Protective Effect by Co-Exposure to Other Metals or Trace Elements

Pre-exposure or co-exposure to other elements such as copper, selenium, zinc, and manganese has a protective effect on Cd toxicity. For copper, the protective effect of co-exposure to Cd has been shown, for example, in mice [151]. However, the cellular mechanism behind this effect remains unknown. For the trace element selenium, several studies have found a wide-spread beneficial effect on antioxidant status and lipid peroxidation *in vivo* when co-exposed or pre-exposed to Cd [152,153,154]. Remarkably, selenium shows similar protective effects on mitochondrial dysfunction as the classical antioxidant NAC in LLC-PK1 cells [155]. Based on a follow-up study, the same authors conclude that selenium reduces oxidative stress-induced mitochondrial apoptosis [156]. Similar results for selenium have been obtained in chicken splenic lymphocytes exposed to Cd [157]. For zinc, *in vivo* studies in rats show direct antioxidant effects which alleviated Cd oxidative stress [158] as well as genotoxicity [159]. In addition, zinc is also known to induce MT in adult zebrafish [13] or in Madin–Darby bovine kidney cells [160]. Similarly, in mice, the protective effect of manganese pre-exposure has been connected to antioxidative effects, induction of MT and protection of Ca^2+^ homeostasis [161].

All things considered, the reduction of Cd-induced oxidative stress may be the main protective effect caused by co-exposure to trace elements and other metals. Additionally, the co-induction of MT represents an important protective function (see Section 2.3). Recent studies have established yet another protective mechanism: By competing with Cd^2+^ uptake via shared transport mechanisms, Mn^2+^ and Zn^2+^ as well as Fe^2+^ and Ca^2+^ can significantly reduce or inhibit the entry of Cd^2+^ [162]. In the following section we focus on the reduced uptake of Cd^2+^ as a protective mechanism.

### 2.8. Protection by Reduced Uptake of Cd

Due to its high hydrophilicity, Cd has to enter cells via active or passive transport proteins such as receptors, transporters and pores or receptor-mediated endocytosis (RME) of Cd^2+^ bound to MT (Cd-MT) [163]. Cd^2+^ often uses uptake routes intended for essential divalent ions such as Ca^2+^, Fe^2+^, Zn^2+^, or Mn^2+^. Consequently, downregulation of transport proteins is an important protective mechanism for cells, especially for long-term resistance against the heavy metal. One approach to study this mechanism is to use Cd-resistant cell lines and to delineate their mode of Cd^2+^ transport because reduced uptake of Cd^2+^ has been shown to be an important feature of Cd-resistant cells. In the case of mouse embryonic cells, this resistance occurs due to a downregulation of transport systems such as the zinc transporter, divalent metal transporter, and voltage-dependent Ca^2+^ channels [145]. According to another study, in MT 1 and 2 knock-out cells, long-term Cd resistance is acquired by downregulation of T-type Ca^2+^ channels [164]. Finally, also for RME of Cd-MT, an important entry pathway of Cd^2+^ in mammalian kidney, studies indicate a protective mechanism by downregulation of kidney cell surface receptors such as cubilin in a rat model with subchronic exposure [165] and megalin in proximal tubule cells [166,167]. Originally, these experiments addressed Cd-induced proteinuria, the impaired reabsorption of proteins from the proximal tubule due to Cd intoxication. Interestingly, this impairment also represents a protective mechanism against additional Cd-MT uptake with obvious organismic trade-offs.

These studies are important examples for the protection of cells against Cd. The variety of different transport systems involved in Cd movement across the cell membrane as shown by several excellent reviews [162,163,168,169] may include many more protective pathways.

### 2.9. Protection through Removal of Cd

The phenomenon of multidrug resistance was first identified in tumor cell lines which developed resistance to chemotherapeutic treatments. Central to this resistance is the induction of multidrug resistance protein 1. Also known as P-glycoprotein (P-gp), this ATP-dependent transmembrane transporter belonging to the ATP-binding cassette (ABC) class of transmembrane proteins is responsible for pumping cytotoxic substances out of the cell. For example, with prolonged exposure time, a study on proximal tubule cells observed a reduction in Cd-associated apoptosis which was due to a four-fold upregulation of the drug efflux pump multidrug resistance P-gp [170]. The signal for the induction of the pump after Cd exposure was transduced via oxygen radicals and could be prevented by antioxidant intervention. As mentioned above, once inside the cell, Cd^2+^ readily binds to thiol groups of GSH. Therefore, when GS-Cd is removed by P-gp, GSH equivalents also leave the cell. In this respect, complexation of GSH with Cd^2+^ and the resulting efflux from the cell might again represent a way of immediate cellular protection with the inevitably adverse long-term effects of lower GSH levels.

Interestingly, a study on Cd-resistant zebrafish cells (ZF4-Cd) connects the cells’ resistance to an upregulation of multidrug resistance-associated protein (MRP) transport activity, higher rates of Cd removal, elevated expression of other ABC class proteins, and increased content of cellular GSH [171]. It is apparent that upregulation of GSH production is a protective mechanism which serves cells not only as an antioxidant but also protects them as a mediator for Cd removal. By blocking GSH synthesis with buthionine sulfoximine (BSO), a study on proximal tubule cells shows that Cd efflux depends on GSH. This study identifies a novel exit route for GSH and GS-Cd in the ABC family member cystic fibrosis conductance regulator (CFTR), a chloride channel. The authors propose a dual response model involving the CFTR in which low Cd intoxication might be resolved by direct removal of GS-Cd. Higher Cd concentrations might lead to severe GSH depletion with decreased ability of the cell to scavenge Cd-induced ROS, ultimately leading to apoptosis [172].

The environmental equivalent to multidrug resistance has been described as multixenobiotic resistance (MXR). This process has predominantly been observed in aquatic organisms where different anthropogenic contaminants are able to induce the P-gp transporter in order to develop a cellular defense mechanism [173,174]. A similar MXR response towards Cd contamination has been found in aquatic mollusks [175,176,177,178] and fish [179]. Natural variation in abiotic factors can also alter Cd-toxicity. This will be addressed in the next section.

### 2.10. Toxicity of Cd by Altered Environmental Factors

A set concentration of Cd in the environment of an organism can greatly vary in its effects under different abiotic conditions such as temperature, oxygen partial pressure, or salinity. For example, a study in Dogwhelk (*Nucella lapillus*) shows that Cd toxicity is positively correlated to temperature. As part of the protective response, metabolism is reduced and higher energy requirements needed for the stress response are met by using internal glycogen stores [82]. In the oyster, Cd damage is also reduced at lower temperatures leading to higher levels of activity of the antioxidative enzyme aconitase [180,181].

This type of response usually involves lower mitochondrial metabolic flux and ATP turnover at lower temperatures, resulting in a weaker toxicological damage in the presence of Cd. As highlighted in Section 2.2, energetic homeostasis is an important prerequisite for successfully handling Cd toxicity. Interestingly, organisms undergoing thermal acclimation respond better to concurrent toxicological challenges [181,182,183,184].

Co-exposure to hypoxia has been shown to increase the tissue accumulation of Cd in freshwater clams (*Corbicula fluminea*) but also to increase protection by MT induction. However, the combined exposure may at best have a compensatory effect on overall viability [176]. The low oxygen tension leads to increased ventilatory activity with the result of enhancing the Cd bioaccumulation rate [185].

Several studies also investigate the impact of ion content and salinity on Cd toxicity. In the gastropod *N. lapillus*, the response to low salinity levels includes altered Cd accumulation and MT expression [186]. Studies on trout gill Cd^2+^ uptake show that hard water (with more Ca^2+^ ions) protects against Cd^2+^ uptake and toxicity [187]. However, a considerable number of studies have found conflicting results for dissolved ions and salinity and a general rule of effect does not apply to different experimental situations. An attempt to include all relevant water chemistry parameters able to interact with metal toxicity has been made for daphnids and fish in the form of the biotic ligand model (BLM) [188]. In green algae, the BLM shows that Cd^2+^ uptake and toxicity are reduced upon calcium, zinc and cobalt exposure; these elements obviously influence Cd toxicity in aquatic environments [189].

Consequently, when using the responses of biomarkers to project Cd intoxication, it is necessary to consider the influence of different abiotic factors [190].

## 3. Experimental Section

### 3.1. Cell Culture

An adherent embryonic fibroblast zebrafish cell line (Z3) [191] was used for exposure experiments. The cells were grown in cell culture flasks to 80% confluency in Leibovitz 15 (L-15, Thermo Fisher Scientific, Carlsbad, CA, USA) complete media supplemented with 15% fetal bovine serum (FBS), l-glutamine, penicillin-streptomycin, and gentamycin. After trypsination, cells were seeded into 96-well plates and left for attachment at 25 °C overnight. The following day cells were washed once with HBSS (Thermo Fisher Scientific) and incubated with 200 µL of the treatment solutions for 18 h followed by a recovery period of 6 h (200 µL of HBSS without treatments).

### 3.2. Treatments

Cells were treated with two different antioxidants NAC (5, 10 mM) (Roth, Karlsruhe, Germany) and l-ascorbic acid (0.05, 0.1 mM) (Roth) as well as in combination with CdCl_2_ (20 µM, 50 µM) (Sigma-Aldrich, St. Louis, MO, USA). All treatments were prepared in sterile HBSS containing Ca^2+^ and Mg^2+^ with pH adjusted to 7.6. We also included controls treated with L-15 complete media (L-15+) and with L-15 media lacking FBS (L-15−).

### 3.3. Cell Density Assay

After one washing step with HBSS, cell density was immediately measured after the recovery period or, for control experiments, after the treatment period using a fluorescent dye (Hoechst 33342) in a plate reader (Victor X4, Perkin Elmer, Waltham, MA, USA) according to standard procedures described previously [192]. For blank correction, the dye solution without cells was used. Absolute cell numbers were calculated according to a previously prepared standard curve. The antioxidant stock and working solutions were freshly prepared prior to each treatment in HBSS (pH 7.6). All experiments were performed using six technical repeats and a minimum of three biological replicates.

### 3.4. Statistical Analysis

Data were normalized to 10,000 cells of the HBSS treatment to overcome seeding-related differences in cell numbers in the biological replicates. Statistical analysis using *t*-tests was, however, performed prior to data normalization. All groups were compared to the HBSS exposure group. Significance level was set to *p* ≤ 0.05. Normalized data were used to reveal the cumulative toxicity of the 50 µM CdCl_2_ and 0.1 mM VC co-exposure compared to the 50 µM CdCl_2_-treated cells.

## 4. Conclusions

Cd is introduced into the environment largely by human activities. On the cellular and organismic levels, several mechanisms can be adopted to cope with Cd and protect against Cd-induced toxicity.

Perhaps the most prominent protection strategy is the prevention of oxidative stress which is one of the major mechanisms by which Cd exerts its toxicity. It can be postulated that many different means able to reduce oxidative stress will also ameliorate Cd-induced toxicity. However, alteration in cellular redox balance can have negative effects like an increased risk of carcinogenesis. Mitochondrial energetic homeostasis is a fundamental requirement for successful Cd defense but long-term countermeasures may depend on a balanced mitochondrial turnover bearing the risk of enhancing anti-apoptotic signaling. The prevention of cellular damage by free Cd^2+^ via metal chelation seems to be a perfect short-term detoxification strategy. Storage and degradation of, e.g., Cd-MT complexes in lysosomes, however, bear the risk of releasing free Cd^2+^ into the cytosol after cell death. ER stress prevention appears to be another highly important protection mechanism in short-term responses to Cd administration. Again, this process potentially leads to carcinogenesis by inducing cell survival pathways. Cytoskeletal rearrangements have also been shown to protect against Cd toxicity, but might also be responsible for carcinogenic transformation. Due to the presence of trade-offs, hormesis or hormesis-like phenomena reducing Cd-induced cellular damage must be critically reviewed, especially when discussing the outcome at an organismic level. Protection via reduced Cd uptake might involve impaired reabsorption. The improved removal of Cd bears the risk of an increased loss of essential proteins leading to negative side-effects.

Antioxidants [193] and Cd chelation [194,195] have been proposed as a therapeutic approach to Cd intoxication. The risk of side-effects should, however, not be underestimated.

It can, therefore, be concluded that Cd protection or Cd detoxification strategies that prevent cellular damage seldom come without trade-offs like, primarily, an increased risk of carcinogenesis. However, an impressive cellular machinery has evolved across the animal kingdom and can be adopted to cope with Cd insult and other anthropogenic stressors in natural habitats.

## References

[1] Järup L., Akesson A. (2009). Current status of Cd as an environmental health problem. Toxicol. Appl. Pharmacol..

[2] Choong G., Liu Y., Templeton D.M. (2014). Interplay of Calcium and cadmium in mediating cadmium toxicity. Chem. Biol. Interact..

[3] Gardarin A., Chédin S., Lagniel G., Aude J.-C., Godat E., Catty P., Labarre J. (2010). Endoplasmic reticulum is a major target of cadmium toxicity in yeast. Mol. Microbiol..

[4] Stohs S.J., Bagchi D. (1995). Oxidative mechanisms in the toxicity of metal ions. Free Radic. Biol. Med..

[5] Valko M., Morris H., Cronin M.T.D. (2005). Metals, toxicity and oxidative stress. Curr. Med. Chem..

[6] Stohs S.J., Bagchi D., Hassoun E., Bagchi M. (2000). Oxidative mechanisms in the toxicity of chromium and cadmium ions. J. Environ. Pathol. Toxicol. Oncol..

[7] Singhal R.K., Anderson M.E., Meister A. (1987). Glutathione, a first line of defense against cadmium toxicity. FASEB J..

[8] Rana S.V.S., Singh R. (2002). Influence of antioxidants on metallothionein-mediated protection in cadmium-fed rats. Biol. Trace Elem. Res..

[9] Chubatsu L.S., Gennari M., Meneghini R. (1992). Glutathione is the antioxidant responsible for resistance to oxidative stress in V79 Chinese hamster fibroblasts rendered resistant to cadmium. Chem. Biol. Interact..

[10] Nair A.R., Lee W.-K., Smeets K., Swennen Q., Sanchez A., Thévenod F., Cuypers A. (2015). Glutathione and mitochondria determine acute defense responses and adaptive processes in cadmium-induced oxidative stress and toxicity of the kidney. Arch. Toxicol..

[11] Sakurai A., Nishimoto M., Himeno S., Imura N., Tsujimoto M., Kunimoto M., Hara S. (2005). Transcriptional regulation of thioredoxin reductase 1 expression by cadmium in vascular endothelial cells: Role of NF-E2-related factor-2. J. Cell. Physiol..

[12] Chen J., Shaikh Z.A. (2009). Activation of Nrf2 by cadmium and its role in protection against cadmium-induced apoptosis in rat kidney cells. Toxicol. Appl. Pharmacol..

[13] Arini A., Gourves P.Y., Gonzalez P., Baudrimont M. (2015). Metal detoxification and gene expression regulation after a Cd and Zn contamination: An experimental study on *Danio rerio*. Chemosphere.

[14] Huang Y., Li W., Su Z.-Y., Kong A.-N.T. (2015). The complexity of the Nrf2 pathway: Beyond the antioxidant response. J. Nutr. Biochem..

[15] Ma Q. (2013). Role of Nrf2 in Oxidative Stress and Toxicity. Annu. Rev. Pharmacol. Toxicol..

[16] Liu J., Qu W., Kadiiska M.B. (2009). Role of oxidative stress in cadmium toxicity and carcinogenesis. Toxicol. Appl. Pharmacol..

[17] Cuypers A., Plusquin M., Remans T., Jozefczak M., Keunen E., Gielen H., Opdenakker K., Nair A.R., Munters E., Artois T.J. (2010). Cadmium stress: An oxidative challenge. BioMetals.

[18] Bertin G., Averbeck D. (2006). Cadmium: Cellular effects, modifications of biomolecules, modulation of DNA repair and genotoxic consequences (a review). Biochimie.

[19] Bravard A., Campalans A., Vacher M., Gouget B., Levalois C., Chevillard S., Radicella J.P. (2010). Inactivation by oxidation and recruitment into stress granules of hOGG1 but not APE1 in human cells exposed to sub-lethal concentrations of cadmium. Mutat. Res..

[20] Eybl V., Kotyzová D., Bludovská M. (2004). The effect of curcumin on cadmium-induced oxidative damage and trace elements level in the liver of rats and mice. Toxicol. Lett..

[21] Eybl V., Kotyzova D., Koutensky J. (2006). Comparative study of natural antioxidants—Curcumin, resveratrol and melatonin—In cadmium-induced oxidative damage in mice. Toxicology.

[22] Daniel S., Limson J.L., Dairam A., Watkins G.M., Daya S. (2004). Through metal binding, curcumin protects against lead- and cadmium-induced lipid peroxidation in rat brain homogenates and against lead-induced tissue damage in rat brain. J. Inorg. Biochem..

[23] Rennolds J., Malireddy S., Hassan F., Tridandapani S., Parinandi N., Boyaka P.N., Cormet-Boyaka E. (2012). Curcumin regulates airway epithelial cell cytokine responses to the pollutant cadmium. Biochem. Biophys. Res. Commun..

[24] Baiomy A.A., Mansour A.A. (2015). Genetic and histopathological responses to cadmium toxicity in rabbit’s kidney and liver: Protection by Ginger (*Zingiber officinale*). Biol. Trace Elem. Res..

[25] Abdel Moneim A.E., Bauomy A.A., Diab M.M.S., Shata M.T.M., Al-Olayan E.M., El-Khadragy M.F. (2014). The protective effect of *Physalis peruviana* L. against cadmium-induced neurotoxicity in rats. Biol. Trace Elem. Res..

[26] Argüelles N., Alvarez-González I., Chamorro G., Madrigal-Bujaidar E. (2012). Protective effect of grapefruit juice on the teratogenic and genotoxic damage induced by cadmium in mice. J. Med. Food.

[27] Kumar P., Prasad Y., Patra A.K., Ranjan R., Swarup D., Patra R.C., Pal S. (2009). Ascorbic acid, garlic extract and taurine alleviate cadmium-induced oxidative stress in freshwater catfish (*Clarias batrachus*). Sci. Total Environ..

[28] Cavuşoğlu K., Yapar K., Yalçin E. (2009). Royal jelly (honey bee) is a potential antioxidant against cadmium-induced genotoxicity and oxidative stress in albino mice. J. Med. Food.

[29] Argüelles-Velázquez N., Alvarez-González I., Madrigal-Bujaidar E., Chamorro-Cevallos G. (2013). Amelioration of cadmium-produced teratogenicity and genotoxicity in mice given *Arthrospira maxima* (Spirulina) Treatment. Evid. Based Complement. Altern. Med..

[30] Paniagua-Castro N., Escalona-Cardoso G., Hernández-Navarro D., Pérez-Pastén R., Chamorro-Cevallos G. (2011). Spirulina (*Arthrospira*) protects against cadmium-induced teratogenic damage in mice. J. Med. Food.

[31] Jahangir T., Khan T.H., Prasad L., Sultana S. (2005). Alleviation of free radical mediated oxidative and genotoxic effects of cadmium by farnesol in Swiss albino mice. Redox Rep..

[32] Wang W., Sun Y., Liu J., Wang J., Li Y., Li H., Zhang W., Liao H. (2012). Protective effect of theaflavins on cadmium-induced testicular toxicity in male rats. Food Chem. Toxicol..

[33] Krishnan M., Jayaraj R.L., Jagatheesh K., Elangovan N. (2015). Taxifolin mitigates oxidative DNA damage *in vitro* and protects zebrafish (*Danio rerio*) embryos against cadmium toxicity. Environ. Toxicol. Pharmacol..

[34] Jia Y., Lin J., Mi Y., Zhang C. (2011). Quercetin attenuates cadmium-induced oxidative damage and apoptosis in granulosa cells from chicken ovarian follicles. Reprod. Toxicol..

[35] Vicente-Sánchez C., Egido J., Sánchez-González P.D., Pérez-Barriocanal F., López-Novoa J.M., Morales A.I. (2008). Effect of the flavonoid quercetin on cadmium-induced hepatotoxicity. Food Chem. Toxicol..

[36] Bu T., Mi Y., Zeng W., Zhang C. (2011). Protective effect of quercetin on cadmium-induced oxidative toxicity on germ cells in male mice. Anat. Rec..

[37] Renugadevi J., Prabu S.M. (2010). Cadmium-induced hepatotoxicity in rats and the protective effect of naringenin. Exp. Toxicol. Pathol..

[38] Sakr S.A., Bayomy M.F., El-Morsy A.M. (2015). Rosemary extract ameliorates cadmium-induced histological changes and oxidative damage in the liver of albino rat. J. Basic Appl. Zool..

[39] Choi J.-H., Rhee I.-K., Park K.-Y., Park K.-Y., Kim J.-K., Rhee S.-J. (2003). Action of green tea catechin on bone metabolic disorder in chronic cadmium-poisoned rats. Life Sci..

[40] Jahan S., Khan M., Ahmed S., Ullah H. (2014). Comparative analysis of antioxidants against cadmium induced reproductive toxicity in adult male rats. Syst. Biol. Reprod. Med..

[41] Wang W., He Y., Yu G., Li B., Sexton D.W., Wileman T., Roberts A.A., Hamilton C.J., Liu R., Chao Y. (2015). Sulforaphane protects the liver against CdSe quantum dot-induced cytotoxicity. PLoS ONE.

[42] Su Z.-Y., Shu L., Khor T.O., Lee J.H., Fuentes F., Kong A.-N.T. (2013). A perspective on dietary phytochemicals and cancer chemoprevention: Oxidative stress, Nrf2, and epigenomics. Top. Curr. Chem..

[43] El-Sokkary G.H., Nafady A.A., Shabash E.H. (2010). Melatonin administration ameliorates cadmium-induced oxidative stress and morphological changes in the liver of rat. Ecotoxicol. Environ. Saf..

[44] Kim C.Y., Lee M.J., Lee S.M., Lee W.C., Kim J.S. (1998). Effect of melatonin on cadmium-induced hepatotoxicity in male Sprague–Dawley rats. Tohoku J. Exp. Med..

[45] Pi H., Xu S., Reiter R.J., Guo P., Zhang L., Li Y., Li M., Cao Z., Tian L., Xie J. (2015). SIRT3-SOD2-mROS-dependent autophagy in cadmium-induced hepatotoxicity and salvage by melatonin. Autophagy.

[46] García M.T.A., González E.L.M. (2010). Natural antioxidants protect against cadmium-induced damage during pregnancy and lactation in rats’ pups. J. Food Sci..

[47] Ognjanović B.I., Pavlović S.Z., Maletić S.D., Zikić R.V., Stajn A.S., Radojicić R.M., Saicić Z.S., Petrović V.M. (2003). Protective influence of vitamin E on antioxidant defense system in the blood of rats treated with cadmium. Physiol. Res..

[48] Novelli J., Novelli E.L.B., Manzano M.A., Lopes A.M., Cataneo A.C., Barbosa L.L., Ribas B.O. (2000). Effect of α-tocopherol on superoxide radical and toxicity of cadmium exposure. Int. J. Environ. Health Res..

[49] El-Sokkary G.H., Awadalla E.A. (2011). The protective role of Vitamin C against cerebral and pulmonary damage induced by cadmium chloride in male adult albino rat. Open Neuroendocrinol. J..

[50] Liu T., He W., Yan C., Qi Y., Zhang Y. (2011). Roles of reactive oxygen species and mitochondria in cadmium-induced injury of liver cells. Toxicol. Ind. Health.

[51] Abe T., Yamamura K., Gotoh S., Kashimura M., Higashi K. (1998). Concentration-dependent differential effects of *N*-acetyl-l-cysteine on the expression of HSP70 and metallothionein genes induced by cadmium in human amniotic cells. Biochim. Biophys. Acta.

[52] Odewumi C.O., Badisa V.L.D., Le U.T., Latinwo L.M., Ikediobi C.O., Badisa R.B., Darling-Reed S.F. (2010). Protective effects of *N*-acetylcysteine against cadmium-induced damage in cultured rat normal liver cells. Int. J. Mol. Med..

[53] Wang J., Zhu H., Liu X., Liu Z. (2014). *N*-acetylcysteine protects against cadmium-induced oxidative stress in rat hepatocytes. J. Vet. Sci..

[54] Wispriyono B., Matsuoka M., Igisu H., Matsuno K. (1998). Protection from cadmium cytotoxicity by *N*-acetylcysteine in LLC-PK1 cells. J. Pharmacol. Exp. Ther..

[55] Wu C.-A., Chao Y., Shiah S.-G., Lin W.-W. (2013). Nutrient deprivation induces the Warburg effect through ROS/AMPK-dependent activation of pyruvate dehydrogenase kinase. Biochim. Biophys. Acta.

[56] Nzengue Y., Steiman R., Garrel C., Lefèbvre E., Guiraud P. (2008). Oxidative stress and DNA damage induced by cadmium in the human keratinocyte HaCaT cell line: Role of glutathione in the resistance to cadmium. Toxicology.

[57] Khanna S., Mitra S., Lakhera P.C., Khandelwal S. (2015). *N*-acetylcysteine effectively mitigates cadmium-induced oxidative damage and cell death in Leydig cells *in vitro*. Drug Chem. Toxicol..

[58] Oh S.-H., Lim S.-C. (2006). A rapid and transient ROS generation by cadmium triggers apoptosis via caspase-dependent pathway in HepG2 cells and this is inhibited through *N*-acetylcysteine-mediated catalase upregulation. Toxicol. Appl. Pharmacol..

[59] Odewumi C.O., Latinwo L.M., Ruden M.L., Badisa V.L.D., Fils-Aime S., Badisa R.B. (2015). Modulation of cytokines and chemokines expression by NAC in cadmium chloride treated human lung cells. Environ. Toxicol..

[60] Hu K.-H., Li W.-X., Sun M.-Y., Zhang S.-B., Fan C.-X., Wu Q., Zhu W., Xu X. (2015). Cadmium Induced Apoptosis in MG63 Cells by Increasing ROS, Activation of p38 MAPK and Inhibition of ERK 1/2 Pathways. Cell. Physiol. Biochem..

[61] Hsu T., Huang K.-M., Tsai H.-T., Sung S.-T., Ho T.-N. (2013). Cadmium (Cd)-induced oxidative stress down-regulates the gene expression of DNA mismatch recognition proteins MutS homolog 2 (MSH2) and MSH6 in zebrafish (*Danio rerio*) embryos. Aquat. Toxicol..

[62] Luchese C., Zeni G., Rocha J.B.T., Nogueira C.W., Santos F.W. (2007). Cadmium inhibits δ-aminolevulinate dehydratase from rat lung *in vitro*: Interaction with chelating and antioxidant agents. Chem. Biol. Interact..

[63] Ji Y.-L., Wang Z., Wang H., Zhang C., Zhang Y., Zhao M., Chen Y.-H., Meng X.-H., Xu D.-X. (2012). Ascorbic acid protects against cadmium-induced endoplasmic reticulum stress and germ cell apoptosis in testes. Reprod. Toxicol..

[64] Manna P., Sinha M., Sil P.C. (2009). Taurine plays a beneficial role against cadmium-induced oxidative renal dysfunction. Amino Acids.

[65] Almeida J., Diniz Y., Marques S.F., Faine L., Ribas B., Burneiko R., Novelli E.L. (2002). The use of the oxidative stress responses as biomarkers in Nile tilapia (*Oreochromis niloticus*) exposed to *in vivo* cadmium contamination. Environ. Int..

[66] Geret F., Serafim A., Bebianno M.J. (2003). Antioxidant enzyme activities, metallothioneins and lipid peroxidation as biomarkers in *Ruditapes decussatus*?. Ecotoxicology.

[67] Cossu C., Doyotte A., Jacquin M.C., Babut M., Exinger A., Vasseur P. (1997). Glutathione reductase, selenium-dependent glutathione peroxidase, glutathione levels, and lipid peroxidation in freshwater bivalves, *Unio tumidus*, as biomarkers of aquatic contamination in field studies. Ecotoxicol. Environ. Saf..

[68] Doyotte A. (1997). Antioxidant enzymes, glutathione and lipid peroxidation as relevant biomarkers of experimental or field exposure in the gills and the digestive gland of the freshwater bivalve *Unio tumidus*. Aquat. Toxicol..

[69] Thévenod F., Lee W.-K. (2013). Cadmium and cellular signaling cascades: Interactions between cell death and survival pathways. Arch. Toxicol..

[70] Gobe G., Crane D. (2010). Mitochondria, reactive oxygen species and cadmium toxicity in the kidney. Toxicol. Lett..

[71] Cannino G., Ferruggia E., Luparello C., Rinaldi A.M. (2009). Cadmium and mitochondria. Mitochondrion.

[72] Poteet E., Winters A., Xie L., Ryou M.-G., Liu R., Yang S.-H. (2014). *In vitro* protection by pyruvate against cadmium-induced cytotoxicity in hippocampal HT-22 cells. J. Appl. Toxicol..

[73] Wei X., Qi Y., Zhang X., Qiu Q., Gu X., Tao C., Huang D., Zhang Y. (2014). Cadmium induces mitophagy through ROS-mediated PINK1/Parkin pathway. Toxicol. Mech. Methods.

[74] Pi H., Xu S., Zhang L., Guo P., Li Y., Xie J., Tian L., He M., Lu Y., Li M. (2013). Dynamin 1-like-dependent mitochondrial fission initiates overactive mitophagy in the hepatotoxicity of cadmium. Autophagy.

[75] Belyaeva E.A., Sokolova T.V., Emelyanova L.V., Zakharova I.O. (2012). Mitochondrial electron transport chain in heavy metal-induced neurotoxicity: Effects of cadmium, mercury, and copper. Sci. World J..

[76] Dorta D.J., Leite S., deMarco K.C., Prado I.M.R., Rodrigues T., Mingatto F.E., Uyemura S.A., Santos A.C., Curti C. (2003). A proposed sequence of events for cadmium-induced mitochondrial impairment. J. Inorg. Biochem..

[77] Li M., Xia T., Jiang C.S., Li L.J., Fu J.L., Zhou Z.C. (2003). Cadmium directly induced the opening of membrane permeability pore of mitochondria which possibly involved in cadmium-triggered apoptosis. Toxicology.

[78] Lee W.-K., Bork U., Gholamrezaei F., Thévenod F. (2005). Cd^2+^-induced cytochrome c release in apoptotic proximal tubule cells: Role of mitochondrial permeability transition pore and Ca^2+^ uniporter. Am. J. Physiol. Ren. Physiol..

[79] Xu S., Pi H., Chen Y., Zhang N., Guo P., Lu Y., He M., Xie J., Zhong M., Zhang Y. (2013). Cadmium induced Drp1-dependent mitochondrial fragmentation by disturbing Calcium homeostasis in its hepatotoxicity. Cell Death Dis..

[80] Guo P., Pi H., Xu S., Zhang L., Li Y., Li M., Cao Z., Tian L., Xie J., Li R. (2014). Melatonin Improves mitochondrial function by promoting MT1/SIRT1/PGC-1 α-dependent mitochondrial biogenesis in cadmium-induced hepatotoxicity *in vitro*. Toxicol. Sci..

[81] Kil I.S., Shin S.W., Yeo H.S., Lee Y.S., Park J.-W. (2006). Mitochondrial NADP+-dependent isocitrate dehydrogenase protects cadmium-induced apoptosis. Mol. Pharmacol..

[82] Leung K.M.Y., Taylor A.C., Furness R.W. (2000). Temperature-dependent physiological responses of the dogwhelk *Nucella lapillus* to cadmium exposure. J. Mar. Biol. Assoc. UK.

[83] Yang J., Liu D., He Y., Wang L. (2015). Mitochondrial energy metabolism in the hepatopancreas of freshwater crabs (*Sinopotamon henanense*) after cadmium exposure. Environ. Sci. Process. Impacts.

[84] Chen C.-Y., Zhang S.-L., Liu Z.-Y., Tian Y., Sun Q. (2015). Cadmium toxicity induces ER stress and apoptosis via impairing energy homeostasis in cardiomyocytes. Biosci. Rep..

[85] Andersen O. (1984). Chelation of cadmium. Environ. Health Perspect..

[86] Viarengo A., Burlando B., Ceratto N., Panfoli I. (2000). Antioxidant role of metallothioneins: A comparative overview. Cell. Mol. Biol..

[87] Sato M., Kondoh M. (2002). Recent studies on metallothionein: Protection against toxicity of heavy metals and oxygen free radicals. Tohoku J. Exp. Med..

[88] Cols N., Romero-Isart N., Bofill R., Capdevila M., Gonzàlez-Duarte P., Gonzàlez-Duarte R., Atrian S. (1999). *In vivo* copper- and cadmium-binding ability of mammalian metallothionein beta domain. Protein Eng..

[89] Palacios O., Pagani A., Pérez-Rafael S., Egg M., Höckner M., Brandstätter A., Capdevila M., Atrian S., Dallinger R. (2011). Shaping mechanisms of metal specificity in a family of metazoan metallothioneins: Evolutionary differentiation of mollusc metallothioneins. BMC Biol..

[90] Pinter T.B.J., Irvine G.W., Stillman M.J. (2015). Domain Selection in Metallothionein 1A: Affinity-controlled mechanisms of zinc binding and cadmium exchange. Biochemistry.

[91] Jara-Biedma R., González-Dominguez R., García-Barrera T., Lopez-Barea J., Pueyo C., Gómez-Ariza J.L. (2013). Evolution of metallotionein isoforms complexes in hepatic cells of *Mus musculus* along cadmium exposure. Biometals.

[92] Palacios O., Pérez-Rafael S., Pagani A., Dallinger R., Atrian S., Capdevila M. (2014). Cognate and noncognate metal ion coordination in metal-specific metallothioneins: The *Helix pomatia* system as a model. J. Biol. Inorg. Chem..

[93] Höckner M., Stefanon K., de Vaufleury A., Monteiro F., Pérez-Rafael S., Palacios O., Capdevila M., Atrian S., Dallinger R. (2011). Physiological relevance and contribution to metal balance of specific and non-specific Metallothionein isoforms in the garden snail, *Cantareus aspersus*. Biometals.

[94] Tomas M., Domènech J., Capdevila M., Bofill R., Atrian S. (2013). The sea urchin metallothionein system: Comparative evaluation of the SpMTA and SpMTB metal-binding preferences. FEBS Open Biol..

[95] Egli D., Domènech J., Selvaraj A., Balamurugan K., Hua H., Capdevila M., Georgiev O., Schaffner W., Atrian S. (2006). The four members of the *Drosophila* metallothionein family exhibit distinct yet overlapping roles in heavy metal homeostasis and detoxification. Genes Cells.

[96] Höckner M., Dallinger R., Stürzenbaum S.R. (2011). Nematode and snail metallothioneins. J. Biol. Inorg. Chem..

[97] Domènech J., Bofill R., Tinti A., Torreggiani A., Atrian S., Capdevila M. (2008). Comparative insight into the Zn(II)-, Cd(II)- and Cu(I)-binding features of the protozoan *Tetrahymena pyriformis* MT1 metallothionein. Biochim. Biophys. Acta.

[98] Wang Q., Xu J., Chai B., Liang A., Wang W. (2011). Functional comparison of metallothioneins MTT1 and MTT2 from *Tetrahymena thermophila*. Arch. Biochem. Biophys..

[99] Eckschlager T., Adam V., Hrabeta J., Figova K., Kizek R. (2009). Metallothioneins and cancer. Curr. Protein Pept. Sci..

[100] Pedersen M.Ø., Larsen A., Stoltenberg M., Penkowa M. (2009). The role of metallothionein in oncogenesis and cancer prognosis. Prog. Histochem. Cytochem..

[101] Günther V., Lindert U., Schaffner W. (2012). The taste of heavy metals: Gene regulation by MTF-1. Biochim. Biophys. Acta.

[102] Höckner M., Stefanon K., Schuler D., Fantur R., de Vaufleury A., Dallinger R. (2009). Coping with cadmium exposure in various ways: The two helicid snails *Helix pomatia* and *Cantareus aspersus* share the metal transcription factor-2, but differ in promoter organization and transcription of their Cd-metallothionein genes. J. Exp. Zool. A Ecol. Genet. Physiol..

[103] Höckner M., Dallinger R., Stürzenbaum S.R. (2015). Metallothionein gene activation in the earthworm (*Lumbricus rubellus*). Biochem. Biophys. Res. Commun..

[104] Freisinger E., Vašák M. (2013). Cadmium in metallothioneins. Met. Ions Life Sci..

[105] Langelueddecke C., Lee W.-K., Thévenod F. (2014). Differential transcytosis and toxicity of the hNGAL receptor ligands cadmium-metallothionein and cadmium-phytochelatin in colon-like Caco-2 cells: Implications for *in vivo* cadmium toxicity. Toxicol. Lett..

[106] Wolff N.A., Lee W.-K., Thévenod F. (2011). Role of Arf1 in endosomal trafficking of protein-metal complexes and cadmium-metallothionein-1 toxicity in kidney proximal tubule cells. Toxicol. Lett..

[107] Li L., Dong F., Xu D., Du L., Yan S., Hu H., Lobe C.G., Yi F., Kapron C.M., Liu J. (2015). Short-term, low-dose cadmium exposure induces hyperpermeability in human renal glomerular endothelial cells. J. Appl. Toxicol..

[108] Onodera A., Tani M., Michigami T., Yamagata M., Min K.-S., Tanaka K., Nakanishi T., Kimura T., Itoh N. (2012). Role of megalin and the soluble form of its ligand RAP in Cd-metallothionein endocytosis and Cd-metallothionein-induced nephrotoxicity *in vivo*. Toxicol. Lett..

[109] Delalande O., Desvaux H., Godat E., Valleix A., Junot C., Labarre J., Boulard Y. (2010). Cadmium-glutathione solution structures provide new insights into heavy metal detoxification. FEBS J..

[110] Liebeke M., Garcia-Perez I., Anderson C.J., Lawlor A.J., Bennett M.H., Morris C.A., Kille P., Svendsen C., Spurgeon D.J., Bundy J.G. (2013). Earthworms produce phytochelatins in response to arsenic. PLoS ONE.

[111] Hall J., Haas K.L., Freedman J.H. (2012). Role of MTL-1, MTL-2, and CDR-1 in mediating cadmium sensitivity in Caenorhabditis elegans. Toxicol. Sci..

[112] Hirano T., Ueda H., Kawahara A., Fujimoto S. (1991). Cadmium toxicity on cultured neonatal rat hepatocytes: Biochemical and ultrastructural analyses. Histol. Histopathol..

[113] Biagioli M., Pifferi S., Ragghianti M., Bucci S., Rizzuto R., Pinton P. (2008). Endoplasmic reticulum stress and alteration in Calcium homeostasis are involved in cadmium-induced apoptosis. Cell Calcium.

[114] Gorman A.M., Healy S.J.M., Jäger R., Samali A. (2012). Stress management at the ER: Regulators of ER stress-induced apoptosis. Pharmacol. Ther..

[115] Luo B., Lee A.S. (2013). The critical roles of endoplasmic reticulum chaperones and unfolded protein response in tumorigenesis and anti-cancer therapies. Oncogene.

[116] Liu F., Inageda K., Nishitai G., Matsuoka M. (2006). Cadmium induces the expression of Grp78, an endoplasmic reticulum molecular chaperone, in LLC-PK1 renal epithelial cells. Environ. Health Perspect..

[117] Liu J., Wu K.C., Lu Y.-F., Ekuase E., Klaassen C.D. (2013). Nrf2 protection against liver injury produced by various hepatotoxicants. Oxid. Med. Cell. Longev..

[118] Du K., Takahashi T., Kuge S., Naganuma A., Hwang G.-W. (2014). FBXO6 attenuates cadmium toxicity in HEK293 cells by inhibiting ER stress and JNK activation. J. Toxicol. Sci..

[119] Lim S.-C., Hahm K.-S., Lee S.-H., Oh S.-H. (2010). Autophagy involvement in cadmium resistance through induction of multidrug resistance-associated protein and counterbalance of endoplasmic reticulum stress WI38 lung epithelial fibroblast cells. Toxicology.

[120] Gaubin Y., Vaissade F., Croute F., Beau B., Soleilhavoup J.-P., Murat J.-C. (2000). Implication of free radicals and glutathione in the mechanism of cadmium-induced expression of stress proteins in the A549 human lung cell-line. Biochim. Biophys. Acta.

[121] Liu H., He J., Chi C., Shao J. (2014). Differential *HSP70* expression in *Mytilus coruscus* under various stressors. Gene.

[122] Luan W., Li F., Zhang J., Wen R., Li Y., Xiang J. (2010). Identification of a novel inducible cytosolic *Hsp70* gene in Chinese shrimp *Fenneropenaeus chinensis* and comparison of its expression with the cognate *Hsc70* under different stresses. Cell Stress Chaperones.

[123] Boutet I., Tanguy A., Rousseau S., Auffret M., Moraga D. (2003). Molecular identification and expression of heat shock cognate 70 (*HSC70*) and heat shock protein 70 (*HSP70*) genes in the Pacific oyster *Crassostrea gigas*. Cell Stress Chaperones.

[124] Vilaboa N.E., Calle C., Pérez C., de Blas E., García-Bermejo L., Aller P. (1995). cAMP increasing agents prevent the stimulation of heat-shock protein 70 (*HSP70*) gene expression by cadmium chloride in human myeloid cell lines. J. Cell Sci..

[125] Jing J., Liu H., Chen H., Hu S., Xiao K., Ma X. (2013). Acute effect of copper and cadmium exposure on the expression of heat shock protein 70 in the Cyprinidae fish *Tanichthys albonubes*. Chemosphere.

[126] Wang Z., Templeton D.M. (1996). Cellular factors mediate cadmium-dependent actin depolymerization. Toxicol. Appl. Pharmacol..

[127] Go Y.-M., Orr M., Jones D.P. (2013). Actin cytoskeleton redox proteome oxidation by cadmium. Am. J. Physiol. Lung Cell. Mol. Physiol..

[128] Colon Rodriguez I., Negron Berrios J. (2015). Effects of cadmium on epigenetics of cytoskeletal genes in CHO cells. FASEB J..

[129] Templeton D.M., Liu Y. (2013). Effects of cadmium on the actin cytoskeleton in renal mesangial cells. Can. J. Physiol. Pharmacol..

[130] Li W., Kagan H.M., Chou I.N. (1994). Alterations in cytoskeletal organization and homeostasis of cellular thiols in cadmium-resistant cells. Toxicol. Appl. Pharmacol..

[131] Lau A.T.Y., Chiu J.-F. (2007). The possible role of cytokeratin 8 in cadmium-induced adaptation and carcinogenesis. Cancer Res..

[132] Somji S., Garrett S.H., Toni C., Zhou X.D., Zheng Y., Ajjimaporn A., Sens M.A., Sens D. (2011). A Differences in the epigenetic regulation of MT-3 gene expression between parental and Cd^+2^ or As^+3^ transformed human urothelial cells. Cancer Cell Int..

[133] Calabrese E.J. (2008). Converging concepts: Adaptive response, preconditioning, and the Yerkes–Dodson Law are manifestations of hormesis. Ageing Res. Rev..

[134] Hoffmann G.R. (2009). A perspective on the scientific, philosophical, and policy dimensions of hormesis. Dose Response.

[135] Calabrese E.J., Blain R.B. (2011). The hormesis database: The occurrence of hormetic dose responses in the toxicological literature. Regul. Toxicol. Pharmacol..

[136] Calabrese E.J. (2013). Hormetic mechanisms. Crit. Rev. Toxicol..

[137] Chen Y.Y., Zhu J.Y., Chan K.M. (2014). Effects of cadmium on cell proliferation, apoptosis, and proto-oncogene expression in zebrafish liver cells. Aquat. Toxicol..

[138] Damelin L.H., Vokes S., Whitcutt J.M., Damelin S.B., Alexander J.J. (2000). Hormesis: A stress response in cells exposed to low levels of heavy metals. Hum. Exp. Toxicol..

[139] Mohammadi-Bardbori A., Rannug A. (2014). Arsenic, cadmium, mercury and nickel stimulate cell growth via NADPH oxidase activation. Chem. Biol. Interact..

[140] Chowdhury M.J., Pane E.F., Wood C.M. (2004). Physiological effects of dietary cadmium acclimation and waterborne cadmium challenge in rainbow trout: Respiratory, ionoregulatory, and stress parameters. Comp. Biochem. Physiol. C Toxicol. Pharmacol..

[141] Wirth D., Christians E., Li X., Benjamin I.J., Gustin P. (2003). Use of HSF1(−/−) mice reveals an essential role for HSF1 to protect lung against cadmium-induced injury. Toxicol. Appl. Pharmacol..

[142] Zhang Y., Shen G., Yu Y., Zhu H. (2009). The hormetic effect of cadmium on the activity of antioxidant enzymes in the earthworm Eisenia fetida. Environ. Pollut..

[143] Lefcort H., Freedman Z., House S., Pendleton M. (2008). Hormetic effects of heavy metals in aquatic snails: Is a little bit of pollution good?. Ecohealth.

[144] Banjerdkij P., Vattanaviboon P., Mongkolsuk S. (2003). Cadmium-induced adaptive resistance and cross-resistance to zinc in Xanthomonas campestris. Curr. Microbiol..

[145] Fujishiro H., Kubota K., Inoue D., Inoue A., Yanagiya T., Enomoto S., Himeno S. (2011). Cross-resistance of cadmium-resistant cells to manganese is associated with reduced accumulation of both cadmium and manganese. Toxicology.

[146] Sengupta S., Bhattacharyya N.P. (1996). Oxidative stress-induced cadmium resistance in Chinese hamster V79 cells. Biochem. Biophys. Res. Commun..

[147] Mcclure C.D., Zhong W., Hunt V.L., Chapman F.M., Hill F.V., Priest N.K. (2014). Hormesis results in trade-offs with immunity. Evolution.

[148] Ng C.Y.P., Pereira S., Cheng S.H., Adam-Guillermin C., Garnier-Laplace J., Yu K.N. (2015). Combined effects of depleted uranium and ionising radiation on zebrafish embryos. Radiat. Prot. Dosim..

[149] Belaidi E., Beguin P.C., Levy P., Ribuot C., Godin-Ribuot D. (2008). Prevention of HIF-1 activation and iNOS gene targeting by low-dose cadmium results in loss of myocardial hypoxic preconditioning in the rat. Am. J. Physiol. Heart Circ. Physiol..

[150] Jing Y., Liu L.Z., Jiang Y., Zhu Y., Guo N.L., Barnett J., Rojanasakul Y., Agani F., Jiang B.H. (2012). Cadmium increases HIF-1 and VEGF expression through ROS, ERK, and AKT signaling pathways and induces malignant transformation of human bronchial epithelial cells. Toxicol. Sci..

[151] Li D., Katakura M., Sugawara N. (1995). Improvement of acute cadmium toxicity by pretreatment with copper salt. Bull. Environ. Contam. Toxicol..

[152] El-Sharaky A.S., Newairy A.A., Badreldeen M.M., Eweda S.M., Sheweita S.A. (2007). Protective role of selenium against renal toxicity induced by cadmium in rats. Toxicology.

[153] Ognjanović B.I., Marković S.D., Pavlović S.Z., Zikić R.V., Stajn A.S., Saicić Z.S. (2008). Effect of chronic cadmium exposure on antioxidant defense system in some tissues of rats: Protective effect of selenium. Physiol. Res..

[154] Liu L., Yang B., Cheng Y., Lin H. (2015). Ameliorative effects of selenium on cadmium-induced oxidative stress and endoplasmic reticulum stress in the chicken kidney. Biol. Trace Elem. Res..

[155] Zhou Y.-J., Zhang S.-P., Liu C.-W., Cai Y.-Q. (2009). The protection of selenium on ROS mediated-apoptosis by mitochondria dysfunction in cadmium-induced LLC-PK_1_ cells. Toxicol. Vitro.

[156] Wang Y., Wu Y., Luo K., Liu Y., Zhou M., Yan S., Shi H., Cai Y. (2013). The protective effects of selenium on cadmium-induced oxidative stress and apoptosis via mitochondria pathway in mice kidney. Food Chem. Toxicol..

[157] Liu S., Xu F., Yang Z., Li M., Min Y., Li S. (2014). Cadmium-induced injury and the ameliorative effects of selenium on chicken splenic lymphocytes: Mechanisms of oxidative stress and apoptosis. Biol. Trace Elem. Res..

[158] Brzóska M.M., Rogalska J. (2013). Protective effect of zinc supplementation against cadmium-induced oxidative stress and the RANK/RANKL/OPG system imbalance in the bone tissue of rats. Toxicol. Appl. Pharmacol..

[159] Coogan T.P., Bare R.M., Waalkes M.P. (1992). Cadmium-induced DNA strand damage in cultured liver cells: Reduction in cadmium genotoxicity following zinc pretreatment. Toxicol. Appl. Pharmacol..

[160] Zhang D., Liu J., Gao J., Shahzad M., Han Z., Wang Z., Li J., Sjölinder H. (2014). Zinc supplementation protects against cadmium accumulation and cytotoxicity in Madin–Darby bovine kidney cells. PLoS ONE.

[161] Eybl V., Kotyzová D. (2010). Protective effect of manganese in cadmium-induced hepatic oxidative damage, changes in cadmium distribution and trace elements level in mice. Interdiscip. Toxicol..

[162] Himeno S., Yanagiya T., Fujishiro H. (2009). The role of zinc transporters in cadmium and manganese transport in mammalian cells. Biochimie.

[163] Thévenod F. (2010). Catch me if you can! Novel aspects of cadmium transport in mammalian cells. Biometals.

[164] Leslie E.M., Liu J., Klaassen C.D., Waalkes M.P. (2006). Acquired cadmium resistance in metallothionein-I/II(−/−) knockout cells: Role of the T-type Calcium channel Cacnα1G in cadmium uptake. Mol. Pharmacol..

[165] Santoyo-Sánchez M.P., Pedraza-Chaverri J., Molina-Jijón E., Arreola-Mendoza L., Rodríguez-Muñoz R., Barbier O.C. (2013). Impaired endocytosis in proximal tubule from subchronic exposure to cadmium involves angiotensin II type 1 and cubilin receptors. BMC Nephrol..

[166] Gena P., Calamita G., Guggino W.B. (2010). Cadmium impairs albumin reabsorption by down-regulating megalin and ClC5 channels in renal proximal tubule cells. Environ. Health Perspect..

[167] Wolff N.A., Abouhamed M., Verroust P.J., Thévenod F. (2006). Megalin-dependent internalization of cadmium-metallothionein and cytotoxicity in cultured renal proximal tubule cells. J. Pharmacol. Exp. Ther..

[168] Jenkitkasemwong S., Wang C.-Y., Mackenzie B., Knutson M.D. (2012). Physiologic implications of metal-ion transport by ZIP14 and ZIP8. Biometals.

[169] Thévenod F., Wolff N.A. (2016). Iron transport in the kidney: Implications for physiology and cadmium nephrotoxicity. Metallomics.

[170] Thevenod F., Friedmann J.M., Katsen A.D., Hauser I.A. (2000). Up-regulation of multidrug resistance P-glycoprotein via nuclear factor-κB activation protects kidney proximal tubule cells from cadmium- and reactive oxygen species-induced apoptosis. J. Biol. Chem..

[171] Long Y., Li Q., Wang Y., Cui Z. (2011). MRP proteins as potential mediators of heavy metal resistance in zebrafish cells. Comp. Biochem. Physiol. Part C Toxicol. Pharmacol..

[172] L’hoste S., Chargui A., Belfodil R., Duranton C., Rubera I., Mograbi B., Poujeol C., Tauc M., Poujeol P. (2009). CFTR mediates cadmium-induced apoptosis through modulation of ROS level in mouse proximal tubule cells. Free Radic. Biol. Med..

[173] Bard S.M. (2000). Multixenobiotic resistance as a cellular defense mechanism in aquatic organisms. Aquat. Toxicol..

[174] Ferreira M., Costa J., Reis-Henriques M.A. (2014). ABC transporters in fish species: A review. Front. Physiol..

[175] Ivanina A., Sokolova I. (2008). Effect of cadmium exposure on P-glycoprotein expression and activity in eastern oysters, *Crassostrea virginica*. FASEB J..

[176] Legeay A., Achard-Joris M., Baudrimont M., Massabuau J.-C., Bourdineaud J.-P. (2005). Impact of cadmium contamination and oxygenation levels on biochemical responses in the Asiatic clam *Corbicula fluminea*. Aquat. Toxicol..

[177] Achard M. (2004). Induction of a multixenobiotic resistance protein (MXR) in the Asiatic clam *Corbicula fluminea* after heavy metals exposure. Aquat. Toxicol..

[178] Eufemia N.A., Epel D. (2000). Induction of the multixenobiotic defense mechanism (MXR), P-glycoprotein, in the mussel *Mytilus californianus* as a general cellular response to environmental stresses. Aquat. Toxicol..

[179] Zucchi S., Corsi I., Luckenbach T., Bard S.M., Regoli F., Focardi S. (2010). Identification of five partial ABC genes in the liver of the Antarctic fish Trematomus bernacchii and sensitivity of ABCB1 and ABCC2 to Cd exposure. Environ. Pollut..

[180] Sanni B., Cherkasov A., Sokolova I.M. (2007). Mitochondrial aconitase is sensitive to oxidative stress induced by cadmium and elevated temperatures but not protected by uncoupling proteins in eastern oysters *Crassostrea virginica*. FASEB J..

[181] Cherkasov A.S., Biswas P.K., Ridings D.M., Ringwood A.H., Sokolova I.M. (2006). Effects of acclimation temperature and cadmium exposure on cellular energy budgets in the marine mollusk *Crassostrea virginica*: Linking cellular and mitochondrial responses. J. Exp. Biol..

[182] Sokolova I.M. (2004). Cadmium effects on mitochondrial function are enhanced by elevated temperatures in a marine poikilotherm, *Crassostrea virginica* Gmelin (Bivalvia: Ostreidae). J. Exp. Biol..

[183] Ivanina A.V., Taylor C., Sokolova I.M. (2009). Effects of elevated temperature and cadmium exposure on stress protein response in eastern oysters *Crassostrea virginica* (Gmelin). Aquat. Toxicol..

[184] Vergauwen L., Knapen D., Hagenaars A., Blust R. (2013). Hypothermal and hyperthermal acclimation differentially modulate cadmium accumulation and toxicity in the zebrafish. Chemosphere.

[185] Tran D., Boudou A., Massabuau J.-C. (2001). How water oxygenation level influences cadmium accumulation pattern in the Asiatic clam *Corbicula fluminea* : A laboratory and field study. Environ. Toxicol. Chem..

[186] Leung K.M.Y., Svavarsson J., Crane M., Morritt D. (2002). Influence of static and fluctuating salinity on cadmium uptake and metallothionein expression by the dogwhelk *Nucella lapillus* (L.). J. Exp. Mar. Biol. Ecol..

[187] Pascoe D., Evans S.A., Woodworth J. (1986). Heavy metal toxicity to fish and the influence of water hardness. Arch. Environ. Contam. Toxicol..

[188] Niyogi S., Wood C.M. (2004). Biotic ligand model, a flexible tool for developing site-specific water quality guidelines for metals. Environ. Sci. Technol..

[189] Lavoie M., Fortin C., Campbell P.G.C. (2012). Influence of essential elements on cadmium uptake and toxicity in a unicellular green alga: The protective effect of trace zinc and cobalt concentrations. Environ. Toxicol. Chem..

[190] Vidal M.-L., Bassères A., Narbonne J.-F. (2002). Influence of temperature, pH, oxygenation, water-type and substrate on biomarker responses in the freshwater clam *Corbicula fluminea* (Müller). Comp. Biochem. Physiol. C Toxicol. Pharmacol..

[191] Pando M.P., Pinchak A.B., Cermakian N., Sassone-Corsi P. (2001). A cell-based system that recapitulates the dynamic light-dependent regulation of the vertebrate clock. Proc. Natl. Acad. Sci. USA.

[192] Sandbichler A.M., Aschberger T., Pelster B. (2013). A method to evaluate the efficiency of transfection reagents in an adherent zebrafish cell line. BioRes. Open Access.

[193] Brzóska M.M., Borowska S., Tomczyk M. (2015). Antioxidants as a Potential Preventive and Therapeutic Strategy for Cadmium. Curr. Drug Targets.

[194] Ivanova J., Gluhcheva Y., Arpadjan S., Mitewa M. (2014). Effects of cadmium and monensin on renal and cardiac functions of mice subjected to subacute cadmium intoxication. Interdiscip. Toxicol..

[195] Smith S.W. (2013). The role of chelation in the treatment of other metal poisonings. J. Med. Toxicol..

